# 

**Published:** 1890-12

**Authors:** 


### INDEX

TO THE

### HOMŒOPATHIC PHYSICIAN.

### VOLUME X.

PAGE
Acetic acid, . . . . . . . . . . . . . . 440
Acid lac, . . . . . . . . . . . . . . . . 398
Actea racemosa, . . . . . . . . . . 221, 508
Actæa spicata, . . . . . . . . . . . . . 508
Aconite in its Relation to the Throat. Ed. J. Lee, M. D., . . . . . . . . . 131
Aconitia, . . . . . . . . . . . . . . . . 415
Aconitum Napellus . . . 9, 16, 266, 403, 415, 440, 550
Actinomycosis, . . . . . . . . . . . . 330
Address before the Hahnemann Club of Terra Haute. W. H. Baker, M. D., . . . . . . . . . . . . . . . 63
Agaricus muscarius, . 16, 135, 170, 176, 440
Agnus castus, . . . . . . . . . . . . 440
Ailanthus-gland, . . . . . . . . . . . 440
Alcohol, . . . . . . . . . . . . . . . 561
Allen's Encyclopædia and Index, Errors in. E. W. Berridge, M. D., 425, 477
Allen, T. F., M. D. Gonorrhœa and Homœopathy, . . . . . . . . . 548
A Protest, . . . . . . . . . . . . . 205
Allen's, Dr. T. F., High Potency Allopathy. B. Fincke, M. D., . . . . 251
Allen, A Reply to Dr. T. F. Chas. B. Gilbert, M. D., . . . . . . . . . . 255
Allen's Protest: Another Answer to. G. W. Sherbino, M. D.. . . . . . 310
Aloes, . . . . . . . . . 11, 117, 133, 312, 440
Aloes, Notes on the Characteristics of. Walter M. James, M. D., . . . . 229
Alternation, A Plea for . . . . . . . . 181
Alumina. . . . . . . . . . . . . . . 440
Ambra grisea. . . . . . . . . . . . . 440
American Institute of Homœopathy, Notice of Meeting, . . . . . . . . 191
American Institute, Session of 1890, 227
Ammonium carb., . . . . . . 40, 441, 517
Amm. mur., . . . . . . . . . . . . . 441
Anacard. orient., . . . . . . . . . . . 441
Anemone Nemorosa, . . . . . . . . . 281
Angustura. . . . . . . . . . . . . . 441
Annals of Surgery, The Review of, . . 141

PAGE
Annual Report and Election by the Managers of the Woman's Homœopathic Hospital of Penna. E. J. Lee, M. D., . . . . . . . . 70
Answer. Dr. Guernsey's . . . . . . . 293
Antimon-crud, . . . . . . . . . . . 81, 441
Antimon-tart, . . . . . . . . . . . 441, 550
Antipyrin. Geo. H. Clark, M. D., . . 4
Antiseptic Dressing. G. M. Pease, M.D., 427
Antiseptics. Geo. H. Clark, M. D., 52, 337
Apis-mel., . . . 15, 80, 116, 364, 404, 441, 496
Apocyn-cannab., . . . . . . . . . . . 69
Appeal from the Bureau of Clinical Medicine of I. H. A., . . . . . . 239
Aquelegia vulgaris, . . . . . . . . . . 369
Are the Two Schools Alike? Ed. J. Lee, M. D., . . . . . . . . . . . 24
Argent-met., . . . . . . . . . . . . 441
Argentum nitricum, . . . . . . 80, 81, 441
Argentum nitricum—Mental and Nervous Symptoms. W. M. Butler, M. D., . . . . . . . . . . . . . . 36
Argonaut, The. Kent B. Waite, M. D. Notice of. . . . . . . . . . . . . 563
Argument and Reason. Geo. H. Clark, M. D., . . . . . . . . . . . . . 97
Arnica, . . . . . . . 62, 81, 220, 442, 468, 550
Arsenicum in the Vomiting of Pregnancy, . . . . . . . . . . . . . 284
Arsenicum, . 74, 79, 82, 93, 135, 147, 170, 213, 310, 316, 393, 402, 442, 496, 550
Arsenicum metallicum. . . . . . . 117
Artificial Feeding of Infants, The. Geo. H. Clark, M. D., . . . . . . . . 241
Arum-try., . . . . . . . . . . 442, 497, 551
Asafœtida. . . . . . . . . . . . . 442, 479
Asarum-europ, . . . . . . . . . . 117, 442
Ascot, Dr. Filipe. Clinical Notes, . . 66
Ataxia, Progressive Locomotor, . . . 169
Aurum, . . . . . . . . 81, 313, 319, 442, 479

Baker, W. H., M. D. Address before the Hahnemann Club of Terre Haute, . . . . . . . . . . . . . . 63

PAGE
Balch, Edward T., M. D. An Unusual Symptom, . . . . . . . . . . . . . 136
Help Wanted, . . . . . . . . . . . 286
Help Wanted and Received, . . . 419
La Grippe, . . . . . . . . . . . 128, 222
Verifications, . . . . . . . . . . . 223
Ballard, Dr. E. A., Message of Sympathy to, . . . . . . . . . . . 383
Baltimore Family Health Journal, Review of, . . . . . . . . . . . . 525
Baptisia, . . . . . 86, 134, 135, 209, 220, 222
Baryta-carb, . . . . . . . . . 442, 480, 551
Baylies, B. L. B., M. D. Homœopathy and Pathology, . . . . . . . . . 515
Beaman, Chas. P., M. D. Puerperal Convulsions. . . . . . . . . . . 467
Belfield, Dr. Wm. T., Solicits Information. Note, . . . . . . . . . . . 288
Belladonna, . . 26, 45, 47, 80, 82, 89, 90, 176, 276, 277, 303, 312, 443, 466, 517
Benzoic-acid, . . . . . . . . . . . . 443
Berberis-aquifol, . . . . . . . . . . . 554
Berberis-vulg., . . . . . . . . . . 80, 443, 553
Berridge, E. W., M. D. A Confession and a Warning, . . . . . . . . . 39
Dr. J. C. Boardman's Provings and Clinical Cases, . . . . . . . . . 559
Dr. T. F. Allen on Homœopathy, . 421
Errors in Dr. T. F. Allen's Encyclopædia, . . . . . . . . . . . . 425, 477
Poisoning by Antipyrin, . . . . . 89
Provings, . . . . . . . . . . . . . . 320
Reply to "A Synopsis of a Case of Hemorrhoids," . . . . . . . . . 215
Biological Students, Notice to, . . . . 191
Bismuthum, . . . . . . . . . . . . 443, 551
Black Eye, . . . . . . . . . . . . . . . 48
Blunders. Geo. H. Clark, M. D.. . . . 245
Blunder, An Awful. Dr. G. W. Emery, 267
Boardman's Provings and Clinical Cases, Dr. J. C. E. W. Berridge, M. D., . . . . . . . . . . . . . . 559
Bœnninghausen and Lippe. Ed. J. Lee, M. D., . . . . . . . . . . . . 34
Book Notices, . . . 94, 141, 190, 231, 287, 328, 383, 429, 524, 562
Borax, . . . . . . . . . . . . . . . 443, 478
Bovista, . . . . . . . . . . . . . . . 443
British Medicinal Plants. Alfred Heath, M. D., . 279, 367, 415, 508, 553
Bromides, . . . . . . . . . . . . . . 335
Bromine, . . . . . . . . . . . . . . . 550
Bryonia, . . 79, 82, 86, 170, 187, 210, 254, 266, 308, 316, 443, 506, 551, 559
Buffalo. A Synopsis of a Case of Hemorrhoids, . . . . . . . . . . . 83
Bureau of Surgery of I. H. A., . . . . . 240
Butler, Clarence Willard, M. D. Clinical Reports and their Relation to Homœopathy, . . . . . . . . . . 150
Bulter, W. M., M. D. Argentum Nitricum — Mental and Nervous Symptoms. . . . . . . . . . . . 36

Cactus-grand, . . . . . . . . . . . . . 443
Caladium, . . . . . . . . . . 81, 318, 443
Caltha palustris, . . . . . . . . . . 299
Calc-carb., . . . 13, 80, 91, 122, 356, 444, 471
Cal-phos., . . . . . . . . . . . . . . 403, 404
Camphora, . . . . . . . . . . . . . 444
Cannabis Indica, . . . . . . . . . 320, 478
Cannabis sativa, . . . . . . . . . . . . 444
Cantharis, . . . . . . . . . . . . . 444, 480
Capsicum, . . . . . . . . . . . . . . 444
Carbolic Acid Proving, A. C. M. Selfridge, M. D., . . . . . . . . . . 420

PAGE
Carbo-animal, . . . . . . . . . . . 31, 444
Carbo-veg., . . . . . . . . . 79, 119, 316, 354
Carleton, Edmund, M. D. A Reply to Dr. Guernsey's Defence, . . . . . 378
The Ward's Island Difficulty in New York, . . . . . . . . . . . . 272
Carnem, . . . . . . . . . . . . . . . . 304
Cases from Practice of Dr. Oscar Hansen. Copenhagen. . . . . . . . . 401
Case Read at the Meeting of the *Organon* Society, of Boston, December 28th. Wm. P. Wesselhoeft, M. D., . . . . . . . . . . . . . . 10
Causticum, . . . . . . . . 115, 277, 286, 445
Census Bulletins, . . . . . . . . . . 431, 524
Census Bureau, An Appeal from. Note, 334
Cepa, . . . . . . . . . . . . . . . . 445
Cephalalgia, . . . . . . . . . . . . . 82, 222
Chamomilla, . . . . . . . . 128, 445, 544, 550
Change of Number. . . . . . . . . . . 17
Chelidonium . . . . . . . . . . . . . 445
Chemical Lecture Notes. By H. M. Whelpley, M. D. Review of, . . 564
China, . . . . . . . . . . . 80, 283, 391, 445
Chin-sul., . . . . . . . . . . . . . . . 390
Cholera, A Case of Sporadic. L. Hoopes, M. D., . . . . . . . . . . 282
Cholera at Bagdad, . . . . . . . . . . 191
Chorea: Case of. Dr. Oscar Hansen . 402
Cicuta-vir., . . . . . . . . . . . . . 445
Cimicifuga-race, . . . . . . . . . 31, 446, 465
Cina, . . . . . . . . . . . . 362, 446, 520, 550
Cinchona-off, . . . . . . . . . . . . . . 382
Cinnabar, . . . . . . . . . . . . . . . 357
Clark, Geo. H., M. D.
Antipyrin, . . . . . . . . . . . . 4
Antiseptics, . . . . . . . . . . . 52, 337
Argument and Reason, . . . . . . 97
The Artificial Feeding of Infants, . 241
Blunders, . . . . . . . . . . . . . . 245
Chronic or Interstitial Pneumonia, 387
Colleges, Journals, Pharmacies. . 194
The Difference between the Old School and the New, . . . . . . 100
Dr. Dudgeon on High Potencies, . 533
The European Epidemic, . . . . . 1
Grafts, . . . . . . . . . . . . . . . 531
Hahnemannian Homœopathy, . . 53
Heredity of Tuberculosis, . . . . 433
Intermittent Fever Treated by Friction, . . . . . . . . . . . . 103
Intermittent Pulse. . . . . . . . . 4
The International Hahnemannian Association's Last Meeting, . . 337
La Grippe, . . . . . . . . 3, 49, 51, 102
Medicinal Aggravations, . . . . . 481
Medicine an Empirical Art, . . . 193
Microbes, Bacilli, Cocci, . . . . . 53
The Minuteness of the Dose, . . . 102
To Mongrels and Allopaths, . . . 59
Notes from Past Meetings of the Lippe Society, . . . . . 115, 304, 350
Pathology, . . . . . . . . . . . . . 55
The Other Side, . . . . . . . . . . 535
Physiology, . . . . . . . . . . . . 56
Progressive Homœopathy, . . . . 58
Quinine, . . . . . . . . . . . . . 57, 102
Recovery of Dr. P. P. Wells, . . . 388
The Repetition of the Dose, . . . . 291
Scientific Medicines, . . . . . . . 2
Sequelæ, . . . . . . . . . . . . . . 197
Strong Medicine, . . . . . . . . . 57
Surgery the Opprobrium of Medicine, . . . . . . . . . . . . . . 436
Surgical Treatment of Uterine Troubles, . . . . . . . . . . . . . 243

PAGE

Clark, Geo. H., M. D.
The Treatment of Affections of the Heart, . . . . . . . . . . . . . 385
The Unattainable, . . . . . . . . . 101
The Use of Crude Drugs by Professed Homœopaths, . . . . . 289
Wines and Liquors in Medicine, . 51
Clarke, Dr. Wm. B., . . . . . . . . . . 336
Notice of Marriage, . . . . . . . . . 144
Clausen, Daniel W., M. D. Magnesium-phosphoricum and Schussler's Bio-Chemistry., . . . . . . . . . 12
Sanicula—A Caution, . . . . . 284, 380
Clematis-erecta, . . . . . . . . 280, 358, 446
Clinical Cases, Two, Robert Farley, M. D., . . . . . . . . . . . . . . 219
Clinical Cases. F. L. Griffith, M. D., . 185
Clinical Cases. Clarence N. Payne, M. D., . . . . . . . . . . . . . . . 518
Clinical Cases, Reports of. G. M. Pease, M. D., . . . . . . . . . . . . . . . 307
Clinical Cases. G. W. Sherbino, M. D., 47, 133, 219
Clinical Case, A. Wm. Steinrauf, M. D., . . . . . . . . . . . . . . . 184
Clinical Cases. Ella M. Tuttle, M. D., 283
Clinical Cases. W. A. Yingling, M. D., 93, 400
Clinical Notes. Dr. Filipe Ascot, . . . 66
Cocculus, . . . . . . . . . . . . . . 310, 446
Coffea, . . . . . . . . . . . . . . . 446, 478
Coffee: he was fond of, . . . . . . . . 48
Cohen, S. W., M. D. La Grippe, . . . . 87
Coincidences. Walter M. James, M. D., 198
Colchicum-autum, . . . . . . . . . . 446
Colleges, The Teaching of Pure Homœopathy in the. Walter M. James, M. D., . . . . . . . . . . 537
Colleges, Journals, and Pharmacies. Geo. H. Clark, M. D., . . . . . . 194
Colocynthis, . . . . . . . . . . . . . . . 446
Comments upon Dr. Wolff's Case. Geo. W. Sherbino, M. D., . . . . 31
Concordance, Repertory of Characteristics of Materia Medica. Wm. Gentry, M. D., Review of. . 236
Confession and a Warning, A. E. W. Berridge, M. D., . . . . . . . . . 39
Congestion of the Lungs and its Dangers, by Thos. Nichols, M. D., Review of, . . . . . . . . . . . . 96
Conium-mac, . . . . . . . . . 170, 446, 516
Consumption: Its Cause and Nature, By Rollin R. Gregg, M. D. Review of, . . . . . . . . . . . . . 233
Consumption Not Contagious, . . . . 333
Convulsions, A Reportory of. By E. M. Santee, M. D. Review of, . . 563
Corrections, . . . . . . . . . . . 191
Corrector Corrected A Notice of, . . . 238
Correspondence, . . . . . . . . . . . 321
Cough, Winter. Frederic Preston, M. D., . . . . . . . . . . . . . . 286
Cranch, Edward, M. D. Watch the Effect, . . . . . . . . . . . . . . 263
Crocus-sat., . . . . . . . . . . . 82, 446, 552
Croton-tig., . . . . . . . . . . . . . . 446
Crude Drugs by Professed Homœopaths, The Use of. Geo. H. Clark, M. D., . . . . . . . . . . 289
Cuprum, . . . . . 82, 219, 314, 316, 402, 446
Cuprum-acet., . . . . . . . . . . . . 551, 552
Cyclamen, . . . . . . . . . . . . . . 447, 552

Davis, F. S., M. D. The Importance of a Diagnosis, . . . . . . . . . . . . 210

PAGE

Definition of a Homœopathic Physician, The. Walter M. James, M. D., . . . . . . . . . . . . . 5, 103
Development of the Infant, The. A. McNeil, M. D., . . . . . . . . . . . 18
Dever, I., M. D., Syphilis and Gonorrhœa, . . . . . . . . . . . . . . . 513
Diagnosis, The Importance of a. F. S. Davis, M. D., . . . . . . . . . . . 210
Diarrhœa, a Case of. Wm. P. Wesselhœft, M. D., . . . . . . . . . . . 10
Diarrhœa, Chronic, . . . . . . . . . . 309
Difference between the Old School and the New. Geo. H. Clark, M. D., . . . . . . . . . . . . . . . 100
Dietetic Gazette, The. Note of, . . . . 288
Digitalis, . . . . . . . . . . . . 256, 447, 552
Diphtheria, . . . . . . . . . . . 308, 324
Diphtheria. Wm. Steinrauf, M. D., . . 324
Diphtheria and Croup, . . . . . . . . . 172
Diphtheria, Cyanide of Mercury in, . 173
Diphtherine, . . . . . . . . . . . . . 325
Disease of the Eye and Ear, By C. H. Vilas, M. D. Review of, . . . . . 430
Disease and Sickness, By Samuel Swan, M. D. Review of, . . . . . 142
Disinfectants, . . . . . . . . . . . . . 7
Dose, Another Dose about the. S. L. . 538
Dose, The Question of the, . . . . . . 159
Dose, The Repetition of the. Geo. H. Clark, M. D., . . . . . . . . . . . 291
Dr. Imposed on, The . . . . . . . . . 192
Drosera, . . . . . . . . . . . . . . . 86, 447
Drug Pathogenesy, A Plea for the Cyclopœdia of, . . . . . . . . . . . 165
Dudley Pemberton, M. D. The American Institute Session of 1890, . . 226
Dulcamara, . . . . . . . . . . . . . 447, 543
Dunlevy, Rita, M. D. Hermaphroditism Complicated with Extrophy of the Bladder, . . . . . . . . . . 405
Dutton, J. M., M. D. Some Relations of Hysteria, . . . . . . . . . . . 108
Dynamic Forces of the Homœopathic Remedies, the. From Meeting of I. H. A., . . . . . . . . . . . 457
Dysmenorrhœa, . . . . . . . . . . . 309

Eating for Strength. M. L. Holbrook, M. D. Notice of, . . . . . . . . . 95
Eclecticism, . . . . . . . . . . . . . . 6
Eczema. Wm. Steinrauf, M. D., . . . 462
Editorials, . . 1, 49, 97, 145, 193, 241, 289, 337, 385, 433
Ehrmann, Dr. Frederick. In Memoriam, . . . . . . . . . . . . . . . 380
El Consultor Homœopatico. Notice of, . . . . . . . . . . . . . . . . 141
Electricity in the Diseases of Women. By G. Betton Massey, M. D. Review of, . . . . . . . . . . . . 329
Electric Railway as a Sanitary Measure, The, . . . . . . . . . . . . . 332
Electro-Homœopathy, . . . . . . 167, 183
Emery, Dr. G. W. An Awful Blunder, 267
Encyclopedia, Errors in Dr. T. F. Allen's . . . . . . . . . . . 425, 477
Epidemic. The European. Geo. H. Clark, M. D., . . . . . . . . . . 1
Epidemiological. Dr. Kunkel, . . . . 313
Epilepsy, its Pathology and Treatment. Hobart Amory Hare, M. D. Review of, . . . . . . . . . 526
Errors, Fatal. J. H. Jackson, M. D., . 361
Erysipelas. G. M. Pease, M. D., . . . 505
Eupat-perfol., . . . . . . . . . . . 186, 393

PAGE
Euphorbia-off., . . . . . . . . . . . . . 447
Euphrasia-off., . . . . . . . . . . . 447, 552
Evans, L. Hamilton, M. D. The Relation of Drugs to Pregnancy, . . 510
Every Thursday. Notice of, . . . . . 235
Experience, Our Daily. Wm. Steinrauf, M. D., . . . . . . . . . 557
Extract from a Letter of W. M. Thackeray, 1849, . . . . . . . . . . . 191

Family Homœopath, The. E. B. Shuldam, M. D. Review of, . . . . 526
Farley, Robt., M. D. Two Clinical Cases, . . . . . . . . . . . . . . 219
Weakness after Urination, . . . . 522
Fatal Errors. J. H. Jackson, M. D., 301, 361
Ferrum-magneticum, . . . . . . . . 81
Ferrum-met., . . . . . . . . . . . . . 447
Ferrum-phos., . . . . . . . . . . 118, 558
Fifth Annual Report of the Trustees of Westborough Insane Hospital. Notice of, . . . . . . . . . . . . 95
Fincke, B., M. D. Dr. T. F. Allen's "High Potency Allopathy," . . 251
Proving of Saccharum-lactis, . . . 137
Flatulent Dyspepsia. Dr. José Nadal, 128
Fluoric acid, . . . . . . . . . . . . . 559
Fowler, S. Mills, M. D. A Proving of Lachesis, . . . . . . . . . . . . 421
Fun for Doctors, . . . . . . . 192, 336, 528

Gailliard, Dr. Mono-Pharmacy, . . . 438
Gee, W. S., M. D. Dr. Preston's Case in the August Number, . . . . . 413
Gelsemium, . . . . . . 80, 119, 448, 465, 552
German Homœopathic Congress, A. Dr. Alexander Villers, . . . . . 285
Germ Theory and its Relation to Homœopathy, The, . . . . . . . . 161
Gilbert, Chas. B., M. D. "High Potency Allopathy," . . . . . . . 256
A Reply to Dr. T. F. Allen, . . . . 255
Glonoinum, . . . . . . . . . . . . 448, 552
Gonorrhœa and Homœopathy. T. F. Allen, M. D., . . . . . . . . . . 548
Gonorrhœa and Straight Homœopathy. The Routine Treatment of Fresh. Thos. Skinner, M. D., . . . . . 498
Grace Hospital, Detroit, . . . . . . 191, 528
Grafts. Geo. H. Clark, M. D., . . . . . 531
Grafts. W. A. Yingling, M. D., . . . . 520
Graph., . . . . . . . . . . . . . . . . 448
Great Grandfather? Who was Your, . 336
Griffith, F. L., M. D. Clinical Cases, 185
Guaiacum off., . . . . . . . . . . 448, 552
Guernsey's Answer, Dr., . . . . . . . 293
Guernsey's Defence, Dr., . . . . . . . 323
Guernsey's Defence, Dr. A Reply to, Edmund Carleton, M. D., . . . . 377
Guernsey, Egbert, M. D., and Ward's Island Homœopathic Hospital. A. McNeil, M. D., . . . . . . . . 381
Guernsey, Wm. Jefferson, M. D. Verifications, . . . . . . . . . . . . 475
Verifications in the October Number, . . . . . . . . . . . . . . . 519
Guiding Symptoms of our Materia Medica, by C. Hering, M. D. Review of, . . . . . . . . . . . . . . . 329
Gundlach, J. G., M. D. Sanicula Spring Water, . . . . . . . . 379, 426
Gunpowder Stains, . . . . . . . . . . 48

Hahnemann, Autograph Letter of Samuel, . . . . . . . . . . . 492, 493
Hahnemann Club of Terre Haute: Address before. W. H. Baker, M. D., 63
Hahnemannian Homœopathy. Geo. H. Clark, M. D., . . . . . . . . . 53
Hall, John, M. D. Brief Contribution to the Elucidation of Syphilis, . 473
Hall, John, Sr., M. D. Query for the Readers of the Homœopathic Physician, . . . . . . . . . . 140
Hallucinations, A Census of, . . . . . 335
Hamamelis, . . . . . . . . . . . . 44, 448
Hansen, Dr. Oscar. Copenhagen, Cases from Practice, . . . . . . . . . 401
Harper's Magazine. Review of, . . . 141
Heath, Alfred, M. D. British Medicinal Plants, . . 279, 295, 367, 415, 508, 553
Helleborus, . . . . . . . 367, 368, 369, 448
Help Wanted. Edward T. Balch, M. D., . . . . . . . . . . . . . . 286
Help Wanted and Received. Edward T. Balch, M. D., . . . . . . . . . 419
Hemorrhoids, "Reply to." A Synopsis of a Case of. E. W. Berridge, M. D., . . . . . . . . . . . . . . . 215
Henbane, . . . . . . . . . . . . . . . 497
Hepar-sulph., . . . . . . . . . 67, 94, 448
Heart, The Treatment of Affections of the. Geo. H. Clark, M. D., . . . 385
Hermaphroditism Complicated with Extrophy of the Bladder. Rita Dunlevy, M. D., . . . . . . . . . 405
"High Potency Allopathy." Chas. B. Gilbert, M. D., . . . . . . . . . 256
Hitchcock, H., M. D. Homœopathy in New York City, . . . . . . . 45
Homœopathic Controversy, The, . . . 82
Homœopathic Physician Read? What Shall a. Wm. Steinrauf, M. D., 224
Homœopathic Prescription, by M. A. A. Wolff, M. D. Notice of, . . . 237
Homœopathic Therapeutics, by Samuel Lilienthal, M. D. Review of, 287
Homœopathic Treatment of Alcoholism, The. by Dr. Gallavardin. Review of, . . . . . . . . . . . 328
Homœopathy, Clinical Reports in their Relation to Homœopathy. Clarence W. Butler, M. D., . . . . . 150
Homœopathy, A Declaration for Pure, 182
Homœopathy, Dr. T. F. Allen on. E. W. Berridge, M. D., . . . . . . . 421
Homœopathy, A Few Thoughts for the Intelligent, by S. Mills Fowler, M. D. Review of, . . . . . . . 190
Homœopathy in New York City. H. Hitchcock, M. D., . . . . . . . 45
Homœopathy in Pain. Rev. J. K. Mendenhall, . . . . . . . . . . 213
Homœopathy and Pathology. B. L. B. Baylies, M. D., . . . . . . . . . 515
Homœopathy Phrenologically Considered. Geo W. Sherbino, M. D., 46
Homœopathy, Pure, in the Colleges: The Teaching of. Walter M. James, M. D., . . . . . . . 483, 537
Hood, F. C., M. D. The Importance of Choosing the Indicated Remedy in the Minimum Dose, . . 389
Hoopes, L., M. D. A Case of Sporadic Cholera, . . . . . . . . . . . . 282
Hydrastis, . . . . . . . . . . . . . . 448
Hydroa, or Fever Blisters. G. M. Pease, M. D., . . . . . . . . . . 263
Hyoscyamus, . . . . . 47, 82, 287, 448, 497
Hypericum, . . . . . . . . . . . . . 449
Hypochondria, . . . . . . . . . . . . 229

PAGE
Hysteria. Some Relations of. J. M. Dutton, M. D., . . . . . . . . . 108

Ignatia, . . . . . . . . 130, 147, 449, 496, 550
Importance of Choosing Indicated Remedy in Minimum Dose. F. C. Hood, M. D., . . . . . . . . . . 389
Indiana Institute of Homœopathy, . . 325
Indicated Remedy in the Minimum Dose, The Importance of Choosing. F. C. Hood, M. D, . . . . . . 389
Infant Feeding. W. I. Thayer, M. D., 347
Influenza and Common Colds, the Causes, Character, and Treatment of Each, by W. T. Ferrie, M. D. Review of, . . . . . . . 235
In Memoriam. Dr. Frederick Ehrmann, . . . . . . . . . . . . . . . 380
In Memoriam Henry Noah Martin, M. D., . . . . . . . . . . . . . . 45
In Memoriam. L. S. Reynolds, M. D., 139
Insomnia and Neuralgia. . . . . . . . 171
Intermittent Fever Treated by Friction. Geo. H. Clark, M. D., . . 103
Intermittent Pulse. Geo. H. Clark, M. D., . . . . . . . . . . . . . . 4
International Hahnemannian Association, Notice of Meeting, . . . . 238
International Hahnemannian Association, Eleventh Annual Meeting, 338, 457, 540
Summary of Proceedings, . . . . 338
Dynamic Forces of the Homœopathic Remedies, . . . . . . . 457
Displacement of the Uterus, . . . 540
International Hahnemannian Association's Last Meeting, The. Geo. H. Clark, M. D., . . . . . . . . 337
International Hahnemannian Association. T. Dwight-Stowe, M. D., . 139
International Hahnemannian Association, Proceedings of. J. W. Thomson, M. D., . . . . . . . . . 410
International Homœopathic Congress of Paris, The. Walter M. James, M. D., . . . . . . . . . . 145, 155, 157
International Medical Annual and Practitioner's Index for 1890, The. By P. W. Williams, M. D. Review of, . . . . . . . . . . . 234
Introductory. G. H. Clark, M D., . . 1
Iodium, . . . . . . . . . . . 15, 113, 404, 449
Iod-sulph., . . . . . . . . . . . . . . . 73
Ipecac, . . . . . . . . . . . . . . 449, 466
Iritis and Irido-Choroiditis, . . . . . 171
Ischiophagus, An, . . . . . . . . . . . 331

Jackson, J. H., M. D. Fatal Errors, 301, 361
James, Walter M., M. D. Coincidences, . . . . . . . . . . . . . . . . 198
The Definition of a Homœopathic Physician, . . . . . . 5, 103
Dr. Samuel Lilienthal, . . . . . 536
The International Homœopathic Congress of Paris, . . . . . . 145, 155
Dr. Frank Kraft, . . . . . . . . 437
Notes on the Characteristics of Aloes, . . . . . . . . . . . . . 229
The Teaching of Pure Homœopathy in the Colleges, . . . 483, 537
The Ward's Island "Difficulty" in New York, . . . . . . . . . 188, 246
Jarring, . . . . . . . . . . . . . . . 312
Jarring. F. H. Lutze, M. D., . . . . . 382

PAGE
Jealousy, The Insanity of. Prof. Ball, . . . . . . . . . . . . . . . 494
Journal of Balneology, Review of, . . 524
Journals to the Medical Profession, Value of, . . . . . . . . . . . . 335

Kali-bichromicum, . . 16, 356, 398, 449, 472
Kali-carb., . . . . . . . . . 117, 449, 516, 550
Kali-iodid., . . . . . . . . . . . . . . 74, 76
Kali-phos., . . . . . . . . . . . . . . 342
Kansas City Homœopathic College, The. Annette H. Waggoner, M. D., 228
Kate Field's Washington, Notice of, . 527
Kent, J. T., M. D. A Study in Materia Medica, . . . . . . . . . . . . 322
Kraft, Dr. Frank. W. M. James, M. D., . . . . . . . . . . . . . . 437
"Want to Know you Know," . . 359
Kreosotum, . . . . . . . . . . . . 438, 449
Kunkel, Dr. Epidemiological, . . . 313
La Grippe, . . . . . . . . . . . . 316

Lac-can., . . . . . . . . . . . . . 306, 419
Lachesis, A Proving of. S. Mills Fowler, M. D., . . . . . . . . . . . . 421
Lachesis, . . . 81, 82, 276, 277, 476, 496, 551
Lager Beer, . . . . . . . . . . . . . . 561
La Grippe. Dr. Edward T. Balch, 128, 222
La Grippe. Geo. H. Clark, M. D., 3, 49, 102
La Grippe. S. W. Cohen, M. D., . . . 87
La Grippe. Dr. Kunkel, . . . . . . . 316
La Grippe. H. B. Stiles, M. D., . . . 86, 127
La Grippe. S. Swan, M. D., . . . . . 124
La Grippe. W. A. Yingling, M. D., . 286
Laryngismus Stridulus. Dr. Oscar Hansen, . . . . . . . . . . . . . 404
Laryngismus Stridulus, Clinical Case. Ella M. Tuttle, M. D., . . . . . 91
Laurocerasus, . . . . . . . . . . . . 450
Lead Poisoning. Prof. Litten, . . . . 31
Ledum-palustre, . . . . . . . . . . . 81, 450
Lee, E. J., M. D. Aconite in its Relation to the Throat, . . . . . . . . 131
Annual Report and Election by the Managers of the Woman's Homœopathic Hospital of Pennsylvania, . . . . . . . . . . . . . . 70
Are the two Schools Alike? . . . . 24
Bœnninghausen and Lippe . . . . 34
Mark Twain's Tribute to Homœopathy, . . . . . . . . . . . . . 203
A Materia Medica Pura, . . . . . 504
The Vital Necessity for Fresh Air in Consumption, . . . . . . . . 77
Lilienthal, Dr. Samuel. Walter M. James, M. D., . . . . . . . . . . 536
Lilienthal, Dr. Samuel. See "S. L."
Lil-tig., . . . . . . . . . . . . . 312, 543
Lippe Society, Notes from past Meetings of. Geo. H. Clark, M. D., 115, 304, 350, 478
Lippe, Dr. Ad. The Repetition of the Dose. . . . . . . . . . . . . . 199
Lithium-carb., . . . . . . . . . . . . 81
Litten, Prof. Lead Poisoning, . . . . 31
Lutze, F. H., M. D. Duration of Action and Antidotes of the Principal Remedies, . . . . . . . . . . . 439
Jarring, . . . . . . . . . . . . 382
Lutze, Dr. Paul. Hysterical Spasms Cured with Pulsatilla. . . . . . 366
Lycopodium, . . . 79, 118, 134, 198, 278, 301, 351, 359, 364, 450

Magnes-carb., . . . . . . . . . . . 16, 450

PAGE
Magnes-mur., . . . . . . . . . . . . 74, 450
Magnesium-phosphoricum and Schussler's Bio-Chemistry. Daniel W. Clausen, M. D., . . . . . . . . . 12
Magnes-phos., . . . . . . . . . . 14, 132
Malandrinum, . . . . . . . . . . . . . 305
Mania. G. W. Sherbino, M. D., . . . 83
Manganum-aceticum, . . . . . . 450, 479
Mark Twain's Tribute to Homœopathy. E. J. Lee, M. D., . . . . 203
Martin, Henry Noah, M. D. In Memoriam, . . . . . . . . . . . . . . 45
Martin, J. T., M. D. Some Results from the Abuse of Quinine, . . . . . 484
Marum verum, . . . . . . . . . . . . 450
Materia Medica, The Homœopathic, . 163
Materia Medica, A Plan to Reform the, 164
Materia Medica Pura, A. E. J. Lee, M. D., . . . . . . . . . . . . . . . 504
Materia Medica, A Study in. J. T. Kent, M. D., . . . . . . . . . . 322
McNeil, A., M. D.
The Development of the Infant. Translated from the German, . . 18
Egbert Guernsey, M. D., and Ward's Island Homœopathic Hospital, . . . . . . . . . . . . 381
Natrum muriaticum, . . . . . . . 256
The Provings of Natrum muriaticum, . . . . . . . . . . . . . . . 418
The Sycosis of Hahnemann, . . . 294
Ulcers, . . . . . . . . . . . . . . . 471
Medical Topics, by W. A. Chatterton. Notice of. . . . . . . . . . . . 95
Medicine an Empirical Art. G. H. Clark, M. D.. . . . . . . . . . . 193
Medicines Mixed in One Prescription Absurd, . . . . . . . . . . . . . . 179
Medicinal Aggravation. Geo. H. Clark, M. D., . . . . . . . . . . . . . . 481
Medorrhinum, . . . . . . . . . . . . 499
Melilotus, . . . . . . . . . . . . . . 266
Memphis Journal of the Medical Sciences. Review of, . . . . . 142, 287
Mendenhall, Rev. J. K. Homœopathy in Pain, . . . . . . . . . . . . . . 213
Mentagra and Tinea Capitis (Favus), By S. L., . . . . . . . . . . . . . 73
Menyanthes, . . . . . . . . . . . . . 450
Mephitis, . . . . . . . . . . . . . . . 80
Mercurius in Typhoid Fever. Geo. H. Clark, M. D., . . . . . . . . . . . 51
Mercurius, . . . . . . . 350, 451, 457 506, 551
Mercury, . . . . . . . . . 250, 306, 320, 479
Mercurius Corrosivus, . . 118, 172, 361, 451
Merc-iod. and Kali-iod., Some Provings of. Ella M. Tuttle, M. D., . . . . 473
Mercurius solubilis, . . . 313, 356, 500, 514
Mezereum, . . . . . . . . . . . . . . 451
Microbes, Bacilli, Cocci. Geo. H. Clark, M. D., . . . . . . . . . . . 53
Minuteness of the Dose, The. Geo. H. Clark, M. D., . . . . . . . . . 102
Mongrels and Allopaths, To. Geo. H. Clark, M. D., . . . . . . . . . . 59
Mono-Pharmacy. Dr. Gailliard, . . . 438
Morphine? No! No! No! M. A. A. Wolff, M. D., . . . . . . . . . 94
Moschus, . . . . . . . . . . . . . . . 451
Muriatic acid, . . . . . . . . . . . 451

Nadal, Dr. José. Flatulent Dyspepsia, 128
Nash, E. B., M. D. Therapeutics of the Throat, . . . . . . . . . . . . 410
National Magazine, The, . . . . . 142, 288
Natrum, . . . . . . . . . . . . . . . 451
Natrum-muriaticum. A. McNeil, M.D., 256, 418
Natrum-muriaticum, . . . 16, 263, 345, 351, 391, 448, 451, 496, 517
Natrum-nitrosum, Poisoning with, . 469
Natrum-sulph., . . . . . . . . . . . . 558
Neurosis of the Genito-Urinary System in the Male with Sterility and Impotence, The, by Dr. R. Ultzmann. Review of, . . . . . . . 431
New Remedies, The. Edited by James E. Gross, M. D. Review of, . . 238
New York State Medical Society. Note, 192
Niccolum, . . . . . . . . . . . . . . 438
Nitric acid, . . 16, 80, 295, 312, 358, 451, 472, 478, 513
Nitrum, . . . . . . . . . . . . . . . . 451
Nosodes, . . . . . . . . . . . . . . . 305
Notes and Notices, . . 48, 143, 191, 238, 288, 330, 384, 432, 528, 564
Notes from Past Meetings of the Lippe Society, . . . 14, 79, 115, 304, 350, 478
Nothing New. Note, . . . . . . . . . 6
Nuggets. C. Carleton Smith, M. D., . 549
Nuphar-lutea, . . . . . . . . . . . . 554
Nux-moschata, . . . . . . . . . 17, 82, 452
Nux-vom., A Proving of. Ella M. Tuttle, M. D., . . . . . . . . . . . . 523
Nux-vom., . . 121, 130, 170, 177, 186, 199, 221, 301, 354, 390, 452, 459, 468, 558, 559
Nymphæa alba, . . . . . . . . . . . 554

Obstetrics. Wm. Steinrauf, M. D., . . 299
Of the Drug Curative. Dr. P. P. Wells, 7, 60, 104
Oh-don't-ology, . . . . . . . . . . . 336
Ointments and Oleates, by John Shoemaker, M. D. Review of, . . . 562
Oleander, . . . . . . . . . . . . . . 452
Oleum terebinth, . . . . . . . . . . 73
On Mentagra and Tinea Capitis (Favus), 73
Onosmodium, . . . . . . . . . . . . 520
Opium, . . . . . . . . . 82, 170, 351, 452, 468
Oregon, Annual Address of Geo. Wigg, M. D., President of the Homœopathic Society of, . . . . . . . . 370
Other Side, The. Geo. H. Clark, M. D., 535
Ovarian Neuralgia, Left. Dr. Oscar Hansen, . . . . . . . . . . . . . 404
Oxygen, Verified Provings of. S. Swan, M. D., . . . . . . . . . . . . . 397
Oysters, Poisoning by. S. L., . . . . . 470

Papaver dubium, . . . . . . . . . . . 555
Papaver somniferum (Opium), . . . . 555
Paralysis Agitans, . . . . . . . . . . 176
Paris International Homœopathic Congress, Proceedings of, . . . . . . 157
Paris quadrifol, . . . . . . . . . . . 452
Partnership of Dr. Wm. S. Gee and Dr. H. C. Allen, . . . . . . . . . . . 191
Pasteur, M., . . . . . . . . . . . . . 144
Pathology. G. H. Clark, M. D., . . . 55
Payne, Clarence N. Another Criticism of Dr. Preston's Case, . . . . . . 414
Clinical Cases, . . . . . . . . . . 518
Pease, G. M., M. D. Antiseptic Dressing, . . . . . . . . . . . . . . . 427
Erysipelas, . . . . . . . . . . . . 505
Hydroa, or Fever Blisters, . . . . 263
Reports of Clinical Cases, . . . . . 307
People's Health Journal. Notice of, . 564
Perineum, . . . . . . . . . . . . . . 231
Petroleum, . . . . . . . . . . . . . 351, 452
Petroselenum, . . . . . . . . . . . . 16
Philosophy in Homœopathy, by

PAGE
Charles S. Mack, M. D. Review of, . . . . . . . . . . . . . 383
Phosphorus, . . 16, 17, 40, 67, 80, 82, 120, 147, 276, 277, 316, 321, 395, 402, 518, 551
Phos-ac., . . . . . . . . . . . . . . . 453
Physical Diagnosis and Practical Urinalysis, by John E. Clark, M. D. Review of, . . . . . . . . . . . . 525
Physician? What Constitutes a Homœopathic. P. P. Wells, M. D., 148
Physiology. Geo. H. Clark, M. D., . . 56
Phytolacca-decandra, . . . . . 79, 81, 453
Picrotoxin, . . . . . . . . . . . . . . . 117
Platina, . . . . . . . . . . . . . . . . 539
Poisoning by Antipyrin. E. W. Berridge, M. D., . . . . . . . . . . . 89
Poisoning, Lead, . . . . . . . . . . . . 31
Poisoning, Novel Cases of, . . . . . . 469
Poisoning by Oysters, . . . . . . . . . 470
Poisoning by Potatoes, . . . . . . . . . 469
Potatoes, Poisoning by, . . . . . . . . 469
Plantago major, . . . . . . . . . . . . 287
Plants, British Medicinal. Alfred Heath, M. D., . 279, 295, 367, 415, 508, 553
Platina, . . . . . . . . . . . . . . . . 453
Pleuro-Pneumonia, . . . . . . . . . . 307
Plumbum, . . . . . . . . . . . . 321, 453
Pneumonia, . . . . . . . . . . . . . . 307
Pneumonia, Chronic or Interstitial. Geo. H. Clark, M. D., . . . . . . . 387
Podophyllum, . . . . . . . . . . . 351, 453
Polonia officinalis, . . . . . . . . . . . 509
Polypharmacy, or Mixed Prescriptions, 168
Polypharmacy and the Single Remedy, 177
Polytechnic, The. Notice of, . . . . 432
Potencies, Dr. Dudgeon on High. Geo. H. Clark, M. D., . . . . . . . . . 533
Potency, How to Select the Proper. Editors, . . . . . . . . . . . . . . 119
Practical Electricity in Medicine and Surgery, by G. A. Liebig. Review of, . . . . . . . . . . . . . . . . 431
Practical Sanitary and Economical Cooking, by Mrs. Mary Hinman Able. Review of, . . . . . . . . 430
Precaution Against Consumption. Circular Number 20, by Dr. Benjamin Lee. Review of, . . . . . 237
Precautionary Circulars of the State Board of Health. Notice of, . . 384
Pregnancy, The Relation of Drugs to. L. Hamilton Evans, M. D., . . . 510
Prescriptions, Dr. Conan's Defence of Mixed, . . . . . . . . . . . . . . 181
Preston, Frederick, M. D. Winter Cough, . . . . . . . . . . . . . . 286
Preston, Mahlon, M. D. A Case of Syphilis, . . . . . . . . . . . . . 355
Preston's Case in the August Number. Dr., W. S. Gee, M. D., . . . . . . 413
Preston's Case, Another Criticism of. Dr. Clarence N. Payne, M. D., . 414
Prevention of Colds, . . . . . . . . . 48
Proceedings of the International Hahnemannian Association. I. W. Thomson, M. D., . . . . . . . . . 410
Proceedings of the Internation Homœopathic Congress of Paris, . . . 157
Progressive Homœopathy. Geo. H. Clark, M. D., . . . . . . . . . . . 58
Protest, A. T. F. Allen, M. D., . . . . 205
Provings. E. W. Berridge, M. D., . . 320
Proving of Saccharum-lactis. B. Fincke, M. D., . . . . . . . . . . 137
Psychology of Epidemics, The. Note, 143

PAGE
Psorinum, . . . . . . . . . . . . 132, 453
Puerperal Convulsions. Charles P. Beaman, M. D., . . . . . . . . . . . 467
Puerperal Convulsions, A Case. W. A. Yingling, M. D., . . . . . . . . . 464
Pulsatilla Nigricans, . 16, 27, 118, 280, 281, 318, 342, 366, 453, 499, 550, 552
Pulsatilla, Hysterical Spasms cured with. Dr. Paul Lutze, . . . . . 366
Pulte Quarterly, The. Review of, . . 288
Pure Homœopathy in the Colleges, The Teaching of. Walter M. James, M. D., . . . . . . . . 483, 537
Pyrogen, . . . . . . . . . . . . . . 342, 344

Query for the Readers of the Homœopathic Physician. John Hall, Sr., M. D., . . . . . . . . . . . . 140
Quinine, . . . . . . . . . . . . . 124, 480
Quinine. Geo. H. Clark, M. D., . . 57, 102
Quinine, Some Results from the Abuse of. J. T. Martin, M. D., . . . . . 484
Quinine, Abuse of. Wm. Steinrauf, M. D., . . . . . . . . . . . . . . 352

Ranunculaceæ, . . . . . . . . . . 279, 415
Ranunculus acris, . . . . . . . . . . . 297
Ranunculus bulbosus, . . . . . . 298, 453
Ranunculus Ficaria, . . . . . . . . . . 296
Ranunculus Flammula, . . . . . . . . 296
Ranunculus repens, . . . . . . . . . . 296
Ranunculus sceleratus, . . . . . . 295, 454
Ratanhia, . . . . . . . . . . . . . . . 116
Recollections of General Grant, by Geo. W. Childs. Review of, . . 429
Regulation of Travel and Traffic, Penna. State Board of Health, Notice of, Pamphlet, . . . . . . . . . . . . 236
Remedies, Duration of Action and Antidotes of the Principal. F. H. Lutze, M. D., . . . . . . . . . 438
Removals, . . . . 191, 331, 384, 432, 528, 564
Repetition of the Dose, The. Dr. Ad. Lippe, . . . . . . . . . . . . . . 199
Reply to Dr. T. F. Allen. Charles B. Gilbert, M. D., . . . . . . . . . . 255
Reply to Dr. Allen's "Protest," A. P. P. Wells, M. D., . . . . . . . . . 247
Report and Recommendations of International American Conference upon Postal and Cable Communication with Central and South America, Notice of, . . . 527
Report and Recommendations Concerning Sanitary and Quarantine Regulations in Commerce with the American Republics, Notice of, . . . . . . . . . . . . . . . . 527
Revue Homœopathique Français, Notice of, . . . . . . . . . . . 142
Reynolds, L. S., M. D. In Memoriam, 139
Rheum, . . . . . . . . . . . . . . . . 454
Rhododendron, . . . . . . . . . . . . 454
Rhus-tox., . 27, 89, 92, 220, 263, 283, 307, 319, 454, 507, 518
Rumex, . . . . . . . . . . . . . . 93, 276
Rumex crispus, Some Remarks on. C. Carleton Smith, M. D., . . . . . 275
Ruta graveolens, . . . . . . . . . 454, 516

Sabadilla, . . . . . . . . . . 186, 316, 454
Sabina, . . . . . . . . . . . . . . 321, 454
Saccharum-album., . . . . . . . . . . 478
Sambucus, . . . . . . . . . . . . . . . 454
Sanguinaria-can., . . . . . . . . . 219, 454
Sanicula, . . . . . . . . . . . . . . . . 221

PAGE
Sanicula, A Caution. D. W. Clausen, M. D., . . . . . . . . . . . . . 2?4, 380
Sanicula Spring Water. J. G. Gundlach, M. D., . . . . . . . . . . 378, 427
Sanitary Entombment, by Rev. Chas. R. Treat. Review of, . . . . . 94
Sarsaparilla, . . . . . . . . . . . 118, 454
Scientific Medicine. Geo. H. Clark, M. D., . . . . . . . . . . . . . 2
Secale cornut., . . . . 80, 82, 169, 314, 454
Selenium, . . . . . . . . . . . . . . 455
Selfridge, C. M., M. D. A Carbolic Acid Proving, . . . . . . . . . . . . 420
Senega, . . . . . . . . . . . . . . . . 455
Sepia, . . . . . . . . . 27, 223, 455, 516, 542
Sequelæ. Geo. H. Clark, M. D., . . . 197
Sherbino, G. W., M. D. Another Answer to Allen's Protest, . . . 310
Clinical Cases, . . . . . . . . 47, 133, 219
Comments upon Dr. Wolff's Case, 31
Homœopathy Phrenologically Considered, . . . . . . . . . . . . . 46
Mania, . . . . . . . . . . . . . . . 82
Verifications of Medorrhinum, . . 84
Silicea, . . . . . . . . . 16, 27, 44, 318, 455
Simillimum? What is the. M. A. A. Wolff, M. D., . . . . . . . . . . . 208
Skinner, Thomas, M. D. "The Routine Treatment of Fresh Gonorrhœa and Straight Homœopathy," . . . . . . . . . . . . 498
S. L. (Samuel Lilienthal, M. D.) Another Dose about the Dose, . . . 538
Cases from Practice of Dr. Hansen, 401
Epidemiological, . . . . . . . . . . 313
Hysterical Spasms cured with Pulsatilla, . . . . . . . . . . . . 366
Homœopathic Therapeutics, Review of, . . . . . . . . . . . . . . 287
Insanity of Jealousy, . . . . . . 494
La Grippe, . . . . . . . . . . . . 316
Lead Poisoning, . . . . . . . . . 31
Mentagra and Tinea Capitis (Favus), . . . . . . . . . . . . . . . 73
Novel Cases of Poisoning, . . . . 468
Poisoning by Oysters, . . . . . . 470
Poisoning by Potatoes, . . . . . . 469
Small-pox, . . . . . . . . . . . . . . 304
Smith, C. Carleton, M. D. Nuggets, . 549
Some Remarks on Rumex Crispus, . . 275
Snapping-Turtle Baby, A. Note, . . . 144
Sol-nig., . . . . . . . . . . . . . . . 464
Southern Homœopathic Medical College. Note, . . . . . . . . . . 333
Southern Journal of Homœopathy, The Change of Editor. Note, . 240
Spasms, Hysterical, Cured with Pulsatilla. Dr. Paul Lutze, . . . . . 366
Spigelia, . . . . . . . . . . . . . . . 455
Spinal Concussion, by S. V. Clevenger, M. D. Review of, . . . . . 190
Spongia, . . . . . . . . . . . . . . . 455
Squilla, . . . . . . . . . . . . . . . . 455
Stammering, Treatment of. Note, . . 144
Stannum, . . . . . . . . . . . . . . . 455
Staphisagria, . . . . . . . . . . . 177, 456
Statistics of Farms, Homes, and Mortgages, . . . . . . . . . . . . . 333
Steinrauf, Wm., M. D. Abuse of Quinine, . . . . . . . . . . . . 352
A Clinical Case, . . . . . . . . . 184
Diphtheria, . . . . . . . . . . . . 324
Eczema, . . . . . . . . . . . . . . 462
Obstetrics, . . . . . . . . . . . . . 299
Our Daily Experience, . . . . . . 557
Sunstroke—Asthma, . . . . . . . 92

PAGE
Steinrauf, Wm., M. D.
What Shall a Homœopathic Physician Read, . . . . . . . . . . . 224
Stiles, H. B., M. D. La Grippe, . . 86, 127
Stow, T. Dwight, M. D. International Hahnemannian Association, . . . . . . . . . . . . . . 139
Stramonium, . . . . . . 46, 80, 82, 403, 456
Strong Medicine. Geo. H. Clark, M.D., 57
Strontiana, . . . . . . . . . . . . . . 456
Sulphur, . 11, 17, 69, 74, 82, 83, 120, 134, 169, 184, 186, 224, 266, 278, 294, 317, 354, 363, 456, 497, 513, 517
Sulphur 55M. A Funny Symptom. M. A. A. Wolff, M. D., . . . . 407, 501
Sulphuric acid, . . . . . . . . . . . . 456
Sunstroke—Asthma. Wm. Steinrauf, M. D., . . . . . . . . . . . . . 92
Surgery, A Good Subject for, . . . . . 48
Surgery the Opprobrium of Medicine, Geo. H. Clark, M. D., . . . . . . 436
Swan, Samuel, M. D. La Grippe, . . 124
Provings of Syphilinum, . . . . . 318
Verified Provings of Oxygen, . . . 397
Sycosis of Hahnemann, The. A. McNeil, M. D., . . . . . . . . . . . 294
Sycosis, Some Symptoms of, . . . . . 536
Symptoms, The Value of Trivial. Geo. H. Clark, M. D., . . . . . . . 146
Symptoms, the Value of Trivial. G. M. Pease, M. D., . . . . . . . . . 315
Synopsis of a Case of Hemorrhoids, A. Buffalo, . . . . . . . . . . . . . . 83
Syphilis, Brief Contributions to the Elucidation of. John Hall, M. D., 473
Syphilis, A Case of. Mahlon Preston, M. D., . . . . . . . . . . . . . . 355
Syphilis and Gonorrhœa. I. Dever, M. D., . . . . . . . . . . . . . . . 513
Syphilinum, Provings of. Samuel Swan, M. D., . . . . . . . . . . 318

Tabacum, . . . . . . . . . . 170, 321, 456
Taraxacum, . . . . . . . . . . . . . 457
Tarent., . . . . . . . . . . . . . . 69, 177
Teaching of Pure Homœopathy in the Colleges. Walter M. James, M. D., . . . . . . . . . . . . . . 483, 537
Terebinth, . . . . . . . . . . . . . . 456
Thayer, W. I., M. D. Infant Feeding, 347
Therapeutics, The Three Essential Precepts of Homœopathic, . . . . . 179
Therapeutics of Throat. E. B. Nash, M. D., . . . . . . . . . . . . . 410
Thompson, J. W., M. D. Proceedings of the International Hahnemannian Association, . . . . . . . . . 410
Three Years' Experience of Water Purification by Means of Iron, in Anderson's Revolving Iron Purifier, by E. Devonshire. Review of, . . . . . . . . . . . . . . 231
Thrombidium, . . . . . . . . . . . . 117
Thuja, . . . . . . . . . . 133, 137, 305, 457
To Our Correspondents, . . . . . . . 7
Trained Nurse, The. Review of, . . . 96
Transactions of the Homœopathic Medical Society of Maine. Review of, . . . . . . . . . . . . . . 563
Transactions of the Homœopathic Medical Society of New York for 1889, and of Pennsylvania for 1889. Review of. . . . . . . . 235
Treatise on Materia Medica, Pharmacology, Therapeutics. John M. Shoemaker, M. D. Review of, . 95

PAGE
Treatment of Snake Bites. Dr. S. Wier Mitchell's Undignified dig at Homœopathy. Wm. B. Clark, M. D. Review of, . . . . . . . . . 236
Triticum, . . . . . . . . . . . . . . . 561
Tuberculosis, Heredity of. Geo. H. Clark, M. D., . . . . . . . . . . . 433
Tuttle, Ella M., M. D. Laryngismus Stridulus—Clinical Case, . . . . 91
A Proving of Nux-vomica, . . . . 523
Some Provings of Merc-iod. and Kali-iod., . . . . . . . . . . 474
Some Verifications, . . . . . . . . 550
Two Clinical Cases. W. A. Yingling, M. D., . . . . . . . . . . . . . . 93

Ulcers. A. McNeil, M. D., . . . . . . 470
Ulcers, . . . . . . . . . . . . . . . . . 472
Ulcus Ventriculi. Dr. Oscar Hansen, 401
Unattainable, The. Geo. H. Clark, M. D., . . . . . . . . . . . . . . 101
Unusual Symptom, An. Edward T. Balch, M. D., . . . . . . . . . . . 136
Urination, Weakness after. Robert Farley, M. D., . . . . . . . . . 522
Uterine Troubles, Surgical Treatment of. Geo. H. Clark, M. D., . . . 243
Uterus, Displacements of the, . . . . 540

Vaccination, . . . . . . . . . . . . . . 305
Valeriana, . . . . . . . . . . . . . . . 457
Variolinum, . . . . . . . . . . . . . . 305
Veratrum-alb., 80, 82, 118, 214, 283, 313, 316, 457, 519
Verbascum, . . . . . . . . . . . . . . 457
Verifications. E. T. Balch, M.D., . . 223
Verifications. Wm. Jefferson Guernsey, M. D., . . . . . . . . . . . . 475
Verifications of Medorrhinum. G. W. Sherbino, M. D., . . . . . . . . . 84
Verifications in the October Number. Dr. Wm. Jefferson Guernsey, . . 522
Verifications, Some. Ella M. Tutttle, M. D., . . . . . . . . . . . . . . 558
Villers, Dr. Alexander, A German Homœopathic Congress, . . . . 285
Viola-odorata, . . . . . . . . . . . . . 457
Viola-tri., . . . . . . . . . . . . . . . . 457
Vipera acuatica caranita, . . . . . . 119

PAGE
Vital Necessity for Fresh Air in Consumption, The. E. J. Lee, M. D., 77

Waggoner, Annette H., M. D. The Kansas City Homœopathic College, 228
"Want to Know, you Know." Frank Kraft, M. D., . . . . . . . . . . . 358
Ward's Island "Difficulty" in New York, The. Dr. Edmund Carleton, . . . . . . . . . . . . . . . . 272
Ward Island Difficulty in New York, The. Walter M. James, M. D., 188, 246
Watch the Effect. Edward Cranch, M. D., . . . . . . . . . . . . . . 263
Wells, P. P., M. D. A Reply to Dr. Allen's "Protest." . . . . . . . 247
Of the Drug Curative, . . . . 7, 60, 104
What Constitutes a Homœopathic Physician? . . . . . . . . . . . 148
Message of Sympathy from the I. H. A. to the Venerable Dr. Wells, 382
Recovery of, Geo. H. Clark, M. D., 388
Wesselhœft, Wm. P., M.D. A Case Read at the Meeting of the *Organon* Society, of Boston, December 28th, . . . . . . . . . . . . . . 10
What is the Simillimum? M. A. A. Wolff, M. D., . . . . . . . . . . 27, 132
Wigg, Geo., M. D., Annual Address of, 370
Wines and Liquors in Medicine. Geo. H. Clark, M. D., . . . . . . . . 51
Wolff, M. A. A., M. D. A Funny Symptom—Sulphur 55M, . . . . . 407, 501
Morphine? No! No! No! . . . 94
What is the Simillimum? . 27, 132, 208
Women's Hospital, The Annual Report of, . . . . . . . . . . . . . . . 70

Yingling, W. A., M. D. Two Clinical Cases, . . . . . . . . . . . . . . . 93
A Clinical Case, . . . . . . . . . . 400
Grafts, . . . . . . . . . . . . . . . 520
La Grippe, . . . . . . . . . . . . . 286
Puerperal Convulsions, . . . . . . 465

Zincum, . . . . . . . . . . 16, 80, 170, 457
Zingiber, . . . . . . . . . . . . . . . . 118

NOTICE.

***All articles for publication, books for review, business communications, etc., must be sent to Editors, 1125 Spruce Street, Philadelphia.***

***Subscribers will please notice that this Journal will be sent until arrears are paid and it is ordered discontinued.***

